# Extraction, Storage Duration, and Storage Temperature Affect the Activity of Ascorbate Peroxidase, Glutathione Reductase, and Superoxide Dismutase in Rice Tissue

**DOI:** 10.3390/biology8040070

**Published:** 2019-09-24

**Authors:** Julia Hartmann, Folkard Asch

**Affiliations:** Institute of Agricultural Sciences in the Tropics, University of Hohenheim, 70593 Stuttgart, Germany; hartmann.julia@uni-hohenheim.de

**Keywords:** antioxidants, enzyme activity, freeze-drying, *Oryza sativa*, storage form

## Abstract

In experimental plant science, research often faces large numbers of tissue samples resulting from sequential harvests of a larger number of genotypes and treatments combinations. Analyses of biological processes such as enzyme kinetics are often time-consuming or need specific sample preparation procedures before the actual measurements can be done. Time is thus often the critical factor and the possibility to store plant samples either as tissue or as extracts increases the available timeframe for analyses. Biological molecules such as enzymes often change their activities when stored and thus do not reflect the processes occurring in living tissue. We investigated the effect of different storage methods such as freeze-drying, freezing at −20 °C, and freezing at −80 °C on the activity of three enzymes known as antioxidants, namely ascorbate peroxidase, glutathione reductase, and superoxide dismutase from two rice varieties. Varieties differed in enzyme activity in extracts of fresh material from leaf blades, leaf sheaths, and roots. When subjected to different storage methods, there were no differences between varieties, but strong effects of the different storage methods on enzyme activities were found. The effects of the storage methods on enzyme activity strongly differed between extracts from stored tissue samples or extracts stored from freshly sampled material. We propose enzyme-specific storage methods and durations that allow for expanding the window for analyses in large experimental studies involving destructive samplings for enzyme kinetics.

## 1. Introduction

Changes in concentration or activity of enzymes scavenging reactive oxygen species can be employed in the evaluation of oxidative stress severity. The overexpression of superoxide dismutase (SOD) is known to lead to protection against specific stresses [1]. Mittler [2] names SOD and ascorbate peroxidase (APX) as the main reactive oxygen species scavengers and states that glutathione reductase (GR) is responsible for maintaining a ratio of high reduced per oxidized ratio of ascorbic acid and glutathione.

In this respect, measuring the activity of several ROS (reactive oxygen species) scavenging enzymes in experiments related to plant responses under stress conditions, would allow insights in the defense mechanisms plants employ in response to the stress. However, experiments involving several factors, particularly when studying genotype by environment interactions, usually produce a large number of samples creating a bottleneck in sample processing in the laboratory. Thus, in such experiments, enzyme activity is usually not determined because immediate measurements from fresh samples, as often recommended, are impossible with sample numbers exceeding 100 samples per day. For example, Engel et al. [3] state in their introduction, that ROS scavenging enzymes have been proposed to be involved in leaf tissue tolerance against iron toxicity but they did not determine the activity of the enzymes in their experiments. We assume, the reason for this was the large sample number: their experiment comprised 21 varieties, 4 iron treatments, 3 sampling dates, and 4 replications. If only one sample per plant had to be measured, this would already result in 1008 samples.

This problem can only be addressed with a safe and appropriate storage procedure allowing postponing measurements of enzyme activities to a later date. To date, there is no clear understanding of storage procedures (sample condition, temperature, duration) affecting ROS scavenging enzyme activity [4].

The literature describes three general possibilities to store samples for determining enzyme activity: (1) No storage but using the samples as fast as possible fresh from the plant, which is widely practiced [5,6,7,8], especially with small numbers of samples. (2) Frozen samples, samples are shock frozen in liquid nitrogen [9,10] and stored at −20 °C [11,12] or −80 °C [9] for later use. Some papers include the duration in storage (e.g., [4]), but often the duration in storage is not mentioned (e.g., [10,11,12]). There is no agreement in the literature on the validity of these methods. Ice crystal size, chemical parameters, and morphological microstructures are thought to depend on the freezing rate as protein damage is correlated with the surface area of ice crystals and the texture of ice [13]. Roy and Gupta [14] found that fine ice crystals, as they occur during fast freezing, increased the interfacial area and led to denaturation of the proteins, whereas maximum recovery of activity occurred with slow freezing and fast thawing. Lester [15], found no difference in the activity between fresh and frozen samples in melons and spinach when the tissues were maintained in a hydrated state during storage of the plant tissues.

Arakawa et al. [16] suggested using cryoprotectants in the extraction solution of the protein such as polyvinylpyrrolidine (PVP) or higher concentrations of potassium phosphate buffer to protect the enzymes during the freezing and de-freezing process. (3) Freeze-dried, dehydrated samples, and rehydration before enzyme activity measurement, which is often not recommended due to loss of activity of the enzymes [14]. Moura et al. [4] found that freeze-drying reduced the antioxidant enzymatic activity in sugarcane leaves and stated that it might cause unfolding of the proteins, which lead to irreversible protein denaturation. According to Anchordoquy and Carpenter [17], without PVP, the quaternary structure can be disrupted by freeze-drying.

In addition, samples can be stored either as intact plant tissue to be extracted prior to the activity measurement or they can be stored as extract after the harvest. Storing intact plant tissues is much more common [4,9,10,15] compared to storing extracts [12]. The literature has no information on the effect of sample conditions (intact or extract) on the storability of the sample regarding enzyme activity measurements.

Here we focused on rice tissues, as ROS scavenging enzyme activities are quite often measured in rice tissues [18,19,20], however, little is known about storage procedures (sample condition, temperature, duration) affecting the ROS scavenging enzyme activity in rice [21,22]. Here we compared frozen plant material samples with samples first extracted and then frozen or freeze-dried. Different freezing procedures (at −20 °C, slow freezing, at −80 °C, fast freezing, freeze-drying, fresh samples, and cooled samples) were compared using the same set of samples, once stored as tissue samples, and once stored as extracts. We used two storage durations to evaluate the long-term stability of the enzyme activity of the stored samples.

The aim of this study was to evaluate the influence of different storage methods and length on the activity of antioxidant enzymes in rice plant samples.

## 2. Materials and Methods

All solutions and standards described here were prepared with pro-analysis grade chemicals (obtained from Sigma-Aldrich, Taufkirchen, Germany) in deionized water.

### 2.1. Plant Materials

Seeds of two rice (*Oryza sativa* L.) varieties (IR31785-58-1-2-3-3 (IR31785), and Suakoko8) obtained from AfricaRice, Benin, were pre-germinated for 7 days on water-saturated filter paper in the dark at 28 °C. Sixty seedlings each were transferred into a hydroponic system, consisting of 7 L containers (Eurobox, Auer, Amerang, Germany, 0.6 × 0.4 × 0.15 m) fitted with a rack of PVC-tubes of 4 cm diameter and 12 cm length. Plants were grown in nutrient solution [23], the first week in half strength and afterward in full strength. The nutrient solution was renewed weekly, and the pH was adjusted to pH 5.5 [24].

### 2.2. Extraction and Storage of Extracts and Samples

Four weeks after sowing, for three replications per variety and four plants per replication, the roots and blades and sheaths of the youngest and third youngest fully developed leaf from the main culm were sampled.

The tissue samples were subjected to two different storage methods: (1) storage in the form of extracts and (2) as intact tissues. For the first method, approximately 0.02 g of fresh plant material was extracted directly after harvest with Fast Prep, 60 s, 6.0 m/s (Fast Prep-24, MP Biomedicals, Fisher Scientific GmbH, Schwerte) in 2.0 mL screw-top microtubes (Sarstedt, Taufkirchen, Germany) containing 0.2 g 1.4 mm ceramic beads and six 2.8 mm ceramic beads (Preqlab, VWR, Darmstadt, Germany) for grinding, and 1 mL ice-cold 50 mM phosphate buffer pH 7 containing 1% PVP40 and 0.2 mM EDTA as extraction medium. For the ascorbate peroxidase assay, the extraction medium contained 5 mM ascorbate. Extracts were centrifuged for 5 min at 3 °C at 13,000 rpm (Microfuge, Heraeus Instruments, Hanau, Germany), and supernatants were pooled per replication and aliquoted into eight vials of 0.3 mL each. One aliquot was measured directly for enzyme activities, one aliquot was stored at 4 °C and measured after 24 h, and 3 aliquots each were stored at −20 °C and −80 °C, respectively. For each storage temperature, one aliquot each was measured one week and 12 weeks after extraction. One aliquot stored either at −20 °C or −80 °C was freeze-dried after 48 h (Lyovac GT2, Seib Industrie GmbH, Gernsheim, Germany), stored at room temperature, and enzyme activities were determined 12 weeks after extraction after adding 0.3 mL of deionized water to the dried samples.

For the second method, 0.02 g of fresh plant material of the same sample were weighted in 2.0 mL screw-top vials containing 0.2 g 1.4 mm ceramic beads and six 2.8 mm ceramic beads (Preqlab, VWR, Darmstadt, Germany) for grinding and stored without immediate extraction at −20 °C and −80 °C, respectively. One week and 12 weeks after sampling, the subsamples were extracted as described above and measured for enzyme activities. One subsample each stored at −20 °C and at −80 °C respectively was freeze-dried after 48 h and stored at room temperature. Samples were rehydrated with 1 mL of the extraction buffer 12 weeks after sampling, extracted as described above, and measured for enzyme activities.

### 2.3. Determination of Enzyme Activities

All methods for the determination of the three enzyme activities were adjusted from the originally published methods to facilitate the use of 96 well plates and a plate reader.

Ascorbate peroxidase (APX) activity was determined according to Nakano and Asada [25]. Of the reaction mixture, containing 50 mM potassium phosphate buffer, pH 7.0, 0.2 mM ascorbate, and 0.2 mM hydrogen peroxide in a total volume of 190 µL, 180 µL was pipetted to 10 µL of the sample extract to start the reaction. Absorbance at 290 nm was recorded for 130 s (Infinite M200, Tecan, Männedorf, Switzerland) in 96 well plates (MTP pure grade 96 UV transparent, Brand GmbH und Co KG, Wertheim, Germany). APX activity was calculated according to Maksimovic and Zivanovic [26]; one unit of APX is defined as the amount of enzyme that can oxidize 1 µmol of ascorbic acid per minute. Glutathione reductase (GR) was determined according to Foyer and Halliwell [27]. Of the reaction mixture containing 30 mM potassium phosphate buffer, pH 7.6, 0.2% BSA, 5 mM EDTA, 2.4 mM GSSG, and 0.19 mM NADPH, 160 µL was pipetted to 20 µL sample extract, and the measurement started immediately. Absorbance at 340 nm was recorded for 180 s. GR activity was calculated from the slope of absorbance readings over time based on 1.0 µmol of oxidized glutathione reduced per minute at pH 7.6 at 25 °C defined as one unit (U) (GR Standard G3664, SigmaAldrich, Taufkirchen, Germany).

Superoxide dismutase activity was determined according to Giannopolitis and Ries [28]. Of the reaction mixture containing 0.05 M sodium carbonate, 13 mM methionine, 1.3 µM Riboflavin, and 21 µM nitro blue tetrazolium (NBT) in a total volume of 180 µL, 160 µL was pipetted to 20 µL of the sample extract. The 96 well plate was carefully shaken and placed under a strong light source (LED growth lamp, spLED, Flensburg, Germany) at a distance of 15 cm. After 60 s, initial absorbance was measured at 560 nm. The plate was exposed to the light treatment another 5 min before absorbance was measured again. Since the presence of SOD inhibits the reduction of NBT, the amount of inhibition can be used to quantify the enzyme activity as compared to a standard curve made with a commercially available standard (SOD standard S9697, Sigma Aldrich, Taufkirchen, Germany).

The absorbance of APX and GR was plotted, and only the linear part was used for calculation of the enzyme activity.

## 3. Results

Enzyme activity may be variety-specific. Therefore, in the first section of the results, we present enzyme activity for the individual varieties in response to different storage methods after sampling. In the second section, we focus on the effects of different storage methods on enzyme activity, whereas in the last section, we show the combined effects of storage method and sample conditions during storage on enzyme activity.

### 3.1. Varietal Differences

**Figure 1:**
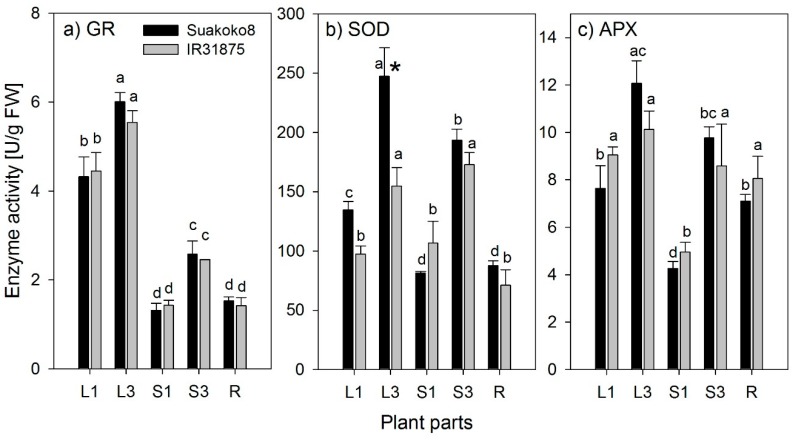
Enzyme activity of (**a**) glutathione reductase (GR), (**b**) superoxide dismutase (SOD), and (**c**) ascorbate peroxidase (APX), measured fresh for 2 varieties, Suakoko8, black, and IR31785, grey, L1 = the leaf blade and S1 = the sheath of the youngest fully developed leaf of the main culm; L3 and S3 = the blade and sheath of the third youngest fully developed leaf of the main culm; R = root. Error bars = standard error of mean (n = 3), asterisk depicts significant differences between varieties, different letters show significant differences between plant parts within the same variety (LSD test, *p* < 0.05).

Figure 1 shows the activities of GR, SOD, and APX in freshly sampled material for different organs of Suakoko8 and IR31785. In general, enzyme activity varied strongly between plant organs and differently so for the different enzymes. However, the organ-specific activity of all measured enzymes did not significantly differ between the varieties with the sole exception of SOD being significantly higher in the older leaf blades of Suakoko8 compared to IR31785. Activities of GR and APX were highest in the leaf blades and tended to be higher in older than in younger plant parts, whereas SOD activities were highest only in older plant parts. The activity in root tissues was relatively low for GR and SOD and at leaf blade level for APX.

**Figure 2:**
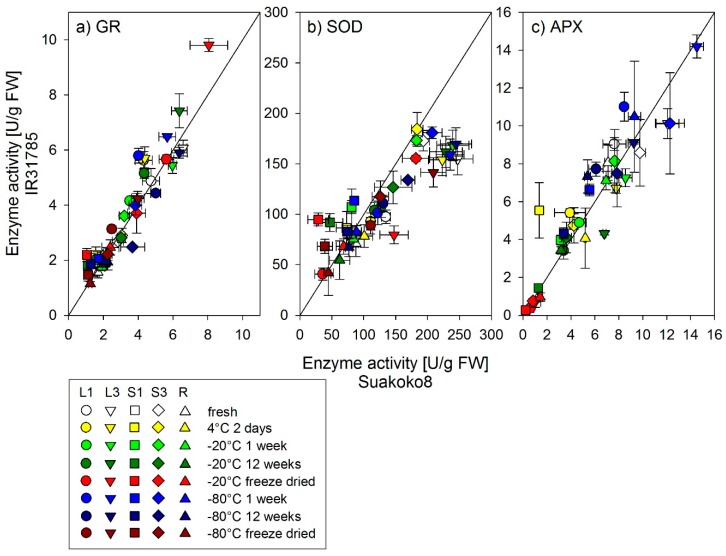
Enzyme activity of (**a**) glutathione reductase (GR), (**b**) superoxide dismutase (SOD), and (**c**) ascorbate peroxidase (APX), for 2 varieties, Suakoko8 and IR31785, L1 = the leaf blade and S1 = the sheath of the youngest fully developed leaf of the main culm; L3 and S3 = the blade and sheath of the third youngest fully developed leaf of the main culm; R = root. Storage treatments are shown in different colors. The line in the subgraphs shows the 1:1 relation. Error bars = standard error of mean (n = 3).

Figure 2 shows the responses of enzyme activities for the three measured enzymes and all plant organs to the different storage treatments as a comparison between the varieties. The comparison with the 1:1 line shows that varieties were not specifically affected by any of the storage treatments. Whereas there are differences between varieties in some individual, organ-specific enzymatic activities, none of the treatments were biased by genotype. Varietal differences are visible for some organs, for example older leaf blades (L3) show higher GR activities in IR31785 as compared to Suakoko8 (Figure 2a), SOD activity in the older leaf blades (L3) is higher for Suakoko8 compared to IR31785, whereas the younger leaf sheaths (S1) have higher activity for IR31785 (Figure 2b). However, these organ-specific differences in enzyme activities were not biased by storage treatment. No varietal differences were observed for APX activity (Figure 2c).

### 3.2. Storing as Intact Tissue or Extract?

**Figure 3:**
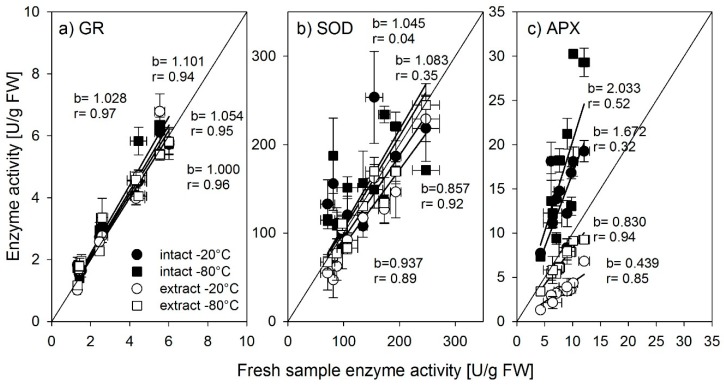
Activity of (**a**) glutathione reductase, (**b**) superoxide dismutase, and (**c**) ascorbate peroxidase determined in fresh samples compared to samples stored as tissue material cut in small pieces. Open symbols: samples stored as extract; filled symbols: intact stored at −20 °C (circles) and −80 °C (squares) and measured 12 weeks after harvest; b depicts the slope of the regression line, r = the correlation coefficient r^2^. Error bars = standard error of mean (n = 3).

Samples can either be extracted prior to storing in the phosphate buffer or can be stored as intact tissues. To compare the effect of the storage form on the enzyme activity, samples of the same tissue were prepared in both ways, and activity was measured 12 weeks after sampling (Figure 3).

Glutathione reductase activity remained basically unchanged after 12 weeks of storage as intact tissue and subsequent extraction (Figure 3a) as well as when stored as frozen extracts for 12 weeks. The activity of superoxide dismutase (Figure 3b) tended to be overestimated in samples stored as intact tissue and underestimated when stored as extracts. Storing temperature did not seem to have a strong influence here. In contrast, ascorbate peroxidase activity (Figure 3c) was strongly influenced by storage temperature (−20 °C always reduced activity as compared to −80 °C) and compared to activities in fresh samples, storage as intact tissue strongly overestimated enzyme activity, whereas, in samples stored as extracts, enzyme activities were strongly reduced compared to fresh samples.

### 3.3. Storage Methods and Duration of Storage

**Figure 4:**
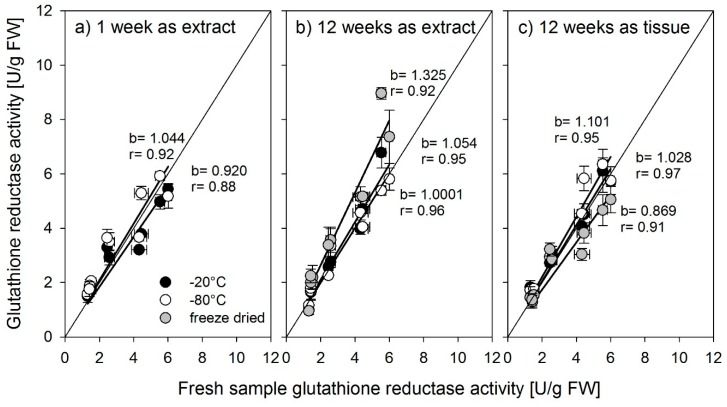
Glutathione reductase activity determined from fresh samples compared with activity determined after storage at −20 °C, (black symbols), −80 °C (open symbols), or freeze-dried extracts of tissue (gray symbols) for (**a**) 1 week storage time or (**b,c**) 12 weeks storage time. b = slope of the regression line, r = the correlation coefficient r^2^. Error bars = standard error of mean (n = 3).

**Figure 5:**
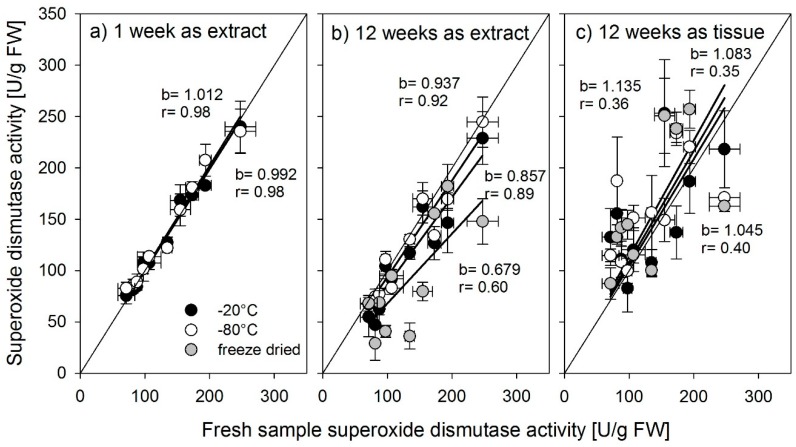
Superoxide dismutase activity determined from fresh samples compared with activity determined after storage at −20 °C, (black symbols), −80 °C (open symbols), or freeze-dried extracts of tissue (gray symbols) for (**a**) 1 week storage time or (**b,c**) 12 weeks storage time. b = slope of the regression line, r = the correlation coefficient r^2^. Error bars = standard error of mean (n = 3).

**Figure 6:**
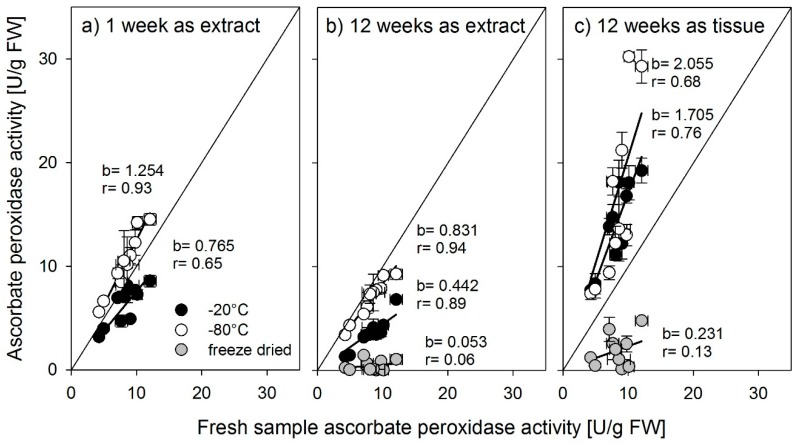
Ascorbate peroxidase activity determined from fresh samples compared with activity determined after storage at −20 °C, (black symbols), −80 °C (open symbols), or freeze-dried extracts of tissue (gray symbols) for (**a**) 1 week storage time or (**b,c**) 12 weeks storage time. b = slope of the regression line, r = the correlation coefficient r^2^. Error bars = standard error of mean (n = 3).

Activities for all three enzymes were determined across organs and varieties from the same samples subjected to different storage methods (stored as tissue or extract), temperatures (−20 °C, −80 °C), and durations (extracts—1 week, extracts and tissue—12 weeks and freeze-dried after harvest) and were compared to their activities in freshly sampled and immediately measured material. A slope close to 1 indicates no effect of the storage treatment on enzyme activities. We show the results for the individual enzymes in Figure 4, Figure 5 and Figure 6.

None of the treatments affected glutathione reductase activity except for freeze-drying (Figure 4). Activities from freeze-dried extracts were overestimated (Figure 4b) whereas activities from freeze-dried tissue were underestimated (Figure 4c). These differences were probably related to the rehydration efficiency of the samples.

Storing extracts at −20 °C or −80 °C for one week did not affect the activity of superoxide dismutase (Figure 5a). Storing extracts or tissue freeze-dried or at −20 °C for 12 weeks severely reduced the activity of superoxide dismutase and increased the inaccuracy of the measurements (Figure 5b,c).

For ascorbate peroxidase, storage strongly affected the activity. Storing for one week as extracts resulted in a strong overestimation when stored at −80 °C and an equally strong underestimation when stored at −20 °C (Figure 6a). Whereas storing extracts at −80 °C for 12 weeks produced the best results for ascorbate peroxidase activity measurements, the activity was generally about 15% lower than in the fresh sample (Figure 6b). Storing tissue samples for 12 weeks resulted in a strong (up to 100%) overestimation of enzyme activities (Figure 6c). Freeze-drying, in general, seemed to have damaged the enzyme since activities after storage were very low or non-existent.

## 4. Discussion

The rice varieties used in the study, IR31785 and Suakoko8, differ in response to iron toxicity [29], thus, differences in the defense to ROS between the genotypes could be expected. However, we did not find any significant differences between the two varieties in the activity of the three enzymes studied here. There were large and significant differences between the different plant organs, but also on an organ level, no differences between the two varieties were found. Minor differences in SOD activity between the two varieties were only found for older leaf blades and were not consistent. Therefore, we discuss the results without varietal aspects. However, the genotypic variation tested here was very small, and our results cannot be interpreted as generally valid for all rice genotypes.

It is quite obvious from the results presented above that freeze-drying samples for later enzyme activity analyses cannot be recommended. In all cases reported here, freeze-drying resulted in enzyme activities strongly different from the activities measured in fresh material. Increased enzyme activities were mainly found in samples that were freeze-dried as tissue samples, which supports the findings from [15] in spinach and watermelon. They explained increases in activity after freeze-drying with the fracturing of organelles and membranes which released more enzymes that were not released during other extraction methods. In samples frozen as extracts, cell organelles are no longer present, since they should be removed in the extraction process. In freeze-dried tissue samples, enzymes from within organelles could be found after rehydration and extraction. In cases where freeze-dried samples resulted in less enzyme activity, either problems with rehydrating the sample may have occurred, or the enzyme was denatured during storage. Anchordoquy and Carpenter [17] suggest that the process of freeze-drying disrupts the quaternary structure of the proteins which may or may not be reversible, while [4] stated that it might cause unfolding of the protein leading to irreversible denaturation.

It could be concluded that leaving the enzymes within the normal cell structure should protect them from conformation changes. We found in most cases that enzyme activities are the same or better-preserved using extracts of plant tissue for storage. Our extraction medium contained 1% PVP, which is known to be an effective cryo-protectant [17]. Cao et al. [13] stated that the freezing rate is of great importance conserving enzyme activity over periods of freezing since the rate of freezing affects the formation of ice crystals. Roy and Gupta [14] found that slow freezing leads to large ice crystals which lead to less denaturation of proteins. As the freezing of plant tissue material consisted of small amounts of cut plant material pieces, we conclude that the freezing of tissue led to faster freezing rates and to denaturation of enzymes, while the comparably bigger amounts of extracts had slower freezing rates.

For simplicity reasons, we suggest storing samples for enzyme activity as tissue samples, which usually does not burden the sampling process and provides a long safe storage period. However, if the sampling procedure and timeframe allow, it may be more beneficial to store samples as extracts even if the process requires to extract the sample and move the supernatant into tubes for freezing. In that case, we suggest keeping the frozen time as short as possible and deep freeze the samples in −80 °C.

Nonetheless, large differences were found between the antioxidant enzymes in the way they were affected by storage. Whereas the activity of glutathione reductase was not affected by storage up to 12 weeks in a frozen state, regardless of storage temperature or form, SOD should be stored as an extract, preferably at −80 °C, or for shorter periods, −20 °C is also possible. Stored at −80 °C, the activity did not decrease up to 12 weeks. Ascorbate peroxidase was most susceptible to storage. Even a short period of one week strongly influenced activity. Lester et al. [15] found a decrease of APX activity in spinach and melon tissue after longer storage, which is confirmed by our findings. For rice tissues, we suggest measuring ascorbate peroxidase activity directly after harvest. However, if storage is needed, the plant extract should be stored at −80 °C for as short a time as possible.

As a fact, the storage duration of samples before measurement of enzyme activity is often not mentioned in literature [4]. We urge reviewers and editors to pay closer attention to such details as subsequent research may come to faulty conclusions if methods are unintentionally compromised.

Plant species also may respond differently to storage in a frozen state. Lester et al. [15], working in dicot species (melon and spinach), states that longer storage of frozen or freeze-dried samples can lead to increased, decreased, or unchanged enzyme activity. In rice, as shown here, enzyme activity mostly decreased with longer storage time.

## 5. Conclusions

The importance of sample storage for enzyme measurements is often underestimated and procedures are often not well documented. The best storage method depends strongly on the enzyme and the sampling procedure. We suggest to measure APX from fresh samples and to freeze intact tissue or extract (depending on the timeframe of the sampling) for SOD and GR.
